# SmMYB4 Is a R2R3-MYB Transcriptional Repressor Regulating the Biosynthesis of Phenolic Acids and Tanshinones in *Salvia miltiorrhiza*

**DOI:** 10.3390/metabo12100968

**Published:** 2022-10-12

**Authors:** Qian Tian, Limin Han, Xiaoya Zhu, Caijuan Zhang, Yunyun Li, Xiaoshan Xue, Yueyue Wang, Donghao Wang, Junfeng Niu, Wenping Hua, Bin Li, Zhezhi Wang

**Affiliations:** 1 Key Laboratory of the Ministry of Education for Medicinal Resources and Natural Pharmaceutical Chemistry, National Engineering Laboratory for Resource Development of Endangered Crude Drugs in Northwest of China, Shaanxi Normal University, Xi’an 710062, China; 2 College of Life Science and Food Engineering, Shaanxi Xueqian Normal University, Xi’an 710062, China; 3 Daan Gene Co., Ltd., Guangzhou 510665, China; 4 Institute of Botany of Shaanxi Province, Xi’an Botanical Garden of Shaanxi Province, Xi’an 710061, China

**Keywords:** *Salvia miltiorrhiza* Bunge, SmMYB4, transcription factor, phenolic acids, tanshinones, transgenic plants

## Abstract

*Salvia miltiorrhiza* Bunge is one of the most famous traditional Chinese medicinal plants. The two most important classes of pharmaceutically relevant compounds in *S. miltiorrhiza* are phenolic acids and tanshinones. The MYB family of transcription factors may efficiently regulate the secondary metabolism in plants. In this study, a subgroup 4 R2R3MYB transcription factor gene, *SmMYB4*, was isolated from *S. miltiorrhiza* and functionally characterized using overexpression and a RNAi-mediated silencing. We achieved a total of six overexpressions and eight RNAi transgenic lines from the *Agrobacterium* leaf disc method. The content of the total phenolics, rosmarinic acid, and salvianolic acid B markedly decreased in the *SmMYB4*-overexpressing lines but increased in the *SmMYB4*-RNAi lines. The content of the total tanshinones, cryptotanshinone, and tanshinone IIA decreased in the *SmMYB4*-overexpressing transgenic lines but increased in the *SmMYB4*-RNAi lines. A gene expression analysis demonstrated that SmMYB4 negatively regulated the transcription of the critical enzyme genes involved in the phenolic acid and tanshinone biosynthesis. The genetic control of this transcriptional repressor may be used to improve the content of these bioactive compounds in the cultivated *S. miltiorrhiza*.

## 1. Introduction

*Salvia miltiorrhiza* Bunge, called ‘Dan-Shen’ in Chinese, is one of the most widely used traditional herbal medicines for various cardiovascular and cerebrovascular diseases. Water-soluble phenolic acids and lipid-soluble tanshinones are the two major classes of pharmaceutical ingredients in *S. miltiorrhiza* [1]. With an increasing market demand for *S. miltiorrhiza*, the wild resources are rapidly decreasing and becoming extremely scarce. However, the quality of cultivated *S. miltiorrhiza* is deteriorating. Therefore, improving the content of the bioactive components in cultivated *S. miltiorrhiza* using modern plant genetic engineering and metabolic engineering technology is important. The biosynthesis, accumulation, and regulation of these active ingredients have become intensively attractive research areas.

The transcription factors (TFs) can bind the cis-elements of a target gene’s promoter region to activate or inhibit its transcription. They offer an effective strategy for enhancing the production of pharmaceutically active metabolites in medicinal plants [2]. Following the publication of the *S. miltiorrhiza* genome [3], many transcription factor gene families, such as MYB, bHLH, WRKY, ERF, and bZIP have been identified and investigated [4,5,6,7,8]. Their important roles in biosynthesis or bioactive ingredient accumulation have been a research focus in recent years. *SmbHLH10*, *SmWRKY1*, and *SmWRKY2* could upregulate the tanshinone biosynthesis [9,10,11]. *SmbHLH51* could positively regulate the biosynthesis of phenolic acids [12]. *SmbHLH148*, *SmbHLH3*, *SmERF115*, and *SmGRAS1/2* not only regulated the biosynthesis of the phenolic acid but also the tanshinone biosynthesis [13,14,15,16].

The MYB transcription factor is one of the largest members of the TF families in plants. Based on the number of imperfect repeats in the DNA-binding domain, the MYB transcription factors are classified into four subfamilies known as 1RMYB, R2R3-MYB, R1R2R3-MYB, and R1R2R3R1/2-MYB. Most MYB proteins in plants are mainly R2R3-MYBs. Various studies have reported that they play critical roles in plant hormone and stress responses, secondary metabolic regulation, organ morphogenesis, and nutrient absorption [4]. *IbMYB116* from the sweet potato enhances drought tolerance in the transgenic *Arabidopsis thaliana* [17]. *MsMYB4* significantly increased the salinity tolerance of alfalfa [18]. The overexpression of *PbrMYB5* in tobacco confers an enhanced tolerance to chilling stresses, whereas the down-regulation of it in the *P. betulaefolia* by a virus-induced gene silencing, results in an elevated chilling sensitivity [19]. In regulating the secondary metabolism, the R2R3-MYB transcription factors have an essential function in producing flavonoid metabolites. MYB1 and AcMYB10 regulate the biosynthesis of anthocyanin in onion and kiwifruit, respectively [20,21]. VrMybA1 and VrMYBCS1 positively correlate with the anthocyanin accumulation in the berry skin [22]. NnMYB5 is a transcription activator of the anthocyanin synthesis contributing to the flower coloration in the lotus (*Nelumbo Adans*) [23]. CcMYB12 and FeMYBF1 regulate the flavonol biosynthesis in the globe artichoke and buckwheat, respectively [24,25]. NtMYB3 represses the biosynthesis of the flavonols in the narcissus and leads to a proanthocyanin accumulation [26]. The MYB transcription factors also modulate the biosynthesis of lignin, chlorophyll, and carotenoid [27,28,29]. Additionally, MYB can regulate the root and leaf development, fruit ripening, Fe homeostasis, and resistance against pathogens [30,31,32,33,34,35,36,37]. With the development of the genome sequencing technology, many species ‘genome data and information have been reported, which provides powerful tools and resources for the identification and in-depth study of the MYB gene family. Now, more and more MYB gene families have been identified from different species, including *Solanum lycopersicum* [38], *Gossypium hirsutum* [39], *Prunus persica* [40], *Solanum tuberosum* [41], *Medicago sativa* [42], and so on. It has become a hot spot to study and understand the function of each member and their mutual relationship.

There are more than 110 R2R3-MYB TFs classified into 33 subgroups in *S. miltiorrhiza* [4], some of which are supposed to regulate the biosynthesis of either the phenolic acids or tanshinones. For instance, SmPAP1 and SmMYB111 are positive regulators of the phenolic acids, while *SmMYB39* negatively regulates their biosynthesis [43,44,45]. *SmMYB36* promotes the tanshinone accumulation but inhibits the phenolic acid biosynthesis [46]. Nevertheless, *SmMYB98* simultaneously improves the accumulation of the tanshinones and salvianolic acids [47]. *SmMYB9b* enhances the tanshinone concentration in hairy roots, too [48]. However, the detailed functions of many other members in the SmMYB TF family remain unknown.

We previously isolated a gene of the subgroup 4 R2R3 MYB, named *SmMYB4* (GU586494.1), from *S. miltiorrhiza* and proposed its possible involvement in the phenolic acid biosynthesis. Herein, we used the overexpression and RNAi-mediated gene silencing to investigate its role in regulating the phenolic acid and tanshinone biosynthesis. The manipulation of the SmMYB4 protein levels offers a potential tool to regulate the metabolic flux responsible for the biosynthesis of the phenolic acids and tanshinones in *S. miltiorrhiza*.

## 2. Materials and Methods

### 2.1. Plant Materials

Mature seeds of *S. miltiorrhiza*, purchased from Shaanxi Tasly Plants Pharmaceutical Co., Ltd. (Shangluo, China), were sewn in a mixed medium containing an equal proportion of vermiculite and nutrient soil and germinated at 25 ± 2 °C, using a cycle of light (16 h at 8000 lx) and dark (8 h). The two-month-old seedlings were frozen in liquid nitrogen for the subsequent total RNA extraction. The seeds were sterilized and cultured on a MS (Murashige and Skoog) medium to obtain sterile plantlets, as described by Yan and Wang [49]. The leaves from these plantlets were used for the genetic transformation experiments.

### 2.2. Subcellular Localization of SmMYB4

The cDNA sequence (with no termination codon) of SmMYB4 was obtained from a public database and then cloned and sequenced from the cDNA library of *S. miltiorrhiza* with the designed primers. The sequence was inserted into pHBT-GFP-NOS vector at KpnI and BamHI restricted sites to form SmMYB4-GFP. The plasmids were transiently transformed into onion epidermal cells using the PDS-1000 Gene Gun (American Bole BIO-RAD, Santa Clara, CA, USA). The onion epidermis cells were isolated and observed under a Leica DM6000B microscope (Leica, Leica Microsystems Co., Ltd., Wetzlar, Germany) at 475 nm.

### 2.3. Construction of the SmMYB4-OE and SmMYB4-RNAi Expression Vectors

According to the manufacturer’s protocol, the total RNA was extracted using E.Z.N.A. OMEGA TM Plant RNA Kit and converted into cDNA using PrimeScript^TM^ RT Master Mix Kit (Takara, Bao Biological Engineering (Dalian) Co., Ltd., Dalian, China).

To construct the overexpression vector, the entire open reading frame (ORF) of *SmMYB4* was amplified with the primers OE*SmMYB4*-*Bgl*II and OE*SmMYB4*-*Bst*PI (Supplementary Material Table S1). The PCR (polymerase chain reaction) products were ligated into the pMD19-T vector (Takara) and transformed into *Escherichia coli* DH5α cells. The recombinant plasmids were digested with *Bgl*II*/Bst*PI and then ligated into the pCAMBIA1302 vector to generate the overexpression plasmid OE*SmMYB4*-1302. To construct the RNAi plasmid, a specific 246 bp fragment of *SmMYB4* was selected as the RNAi target fragment. The forward and reverse RNAi target fragments were amplified with the primers *ISmMYB4-Hind*III/ISmMYB4-*Bam*HI and *ISmMYB4-Kpn*I/*ISmMYB4-Xho*I, respectively, and inserted into the pKANNIBAL vector. An interfering box was cloned into pART27 to generate the *SmMYB4*-RNAi vector named *ISmMYB4*-pART27. To verify whether the overexpression and RNAi vectors were successfully constructed, OE*SmMYB4*-1302 and IS*mMYB4*-pART27 were digested with *Bgl*II /*Bst*PI and *Xho*I/*Not*I, respectively.

### 2.4. Transformation and Detection of S. Miltiorrhiza

The OE*SmMYB4*-1302 and I*SmMYB4*-pART27 vectors were separately transformed into the *Agrobacterium tumefaciens* strain *GV3101* using a freeze-thaw method [50]. The *A. tumefaciens GV3101* harboring the recombinant vectors of OE*SmMYB4*-1302 or I*SmMYB4*-pART27 were used to generate the transgenic plants according to the protocol established in our laboratory [49].

To confirm whether the target sequences had been integrated into the genome of *S. miltiorrhiza*, the genomic DNA was extracted (following OMEGA DNA extraction kit instructions) from the fresh leaves of one-month-old transgenic plantlets and non-transformed (WT) plants. The primers of 35S promoter and Kan resistance gene were used for identifying the overexpression and the RNAi transgenic plantlets, respectively (Supplementary Material Table S2). The PCR products were detected using 1% agarose gel electrophoresis.

The total RNA was isolated from the young leaves and converted into cDNA using the above method. The reverse transcripts were utilized as templates for analyzing the expression of *SmMYB4* in the transgenic lines and the WT lines, using real-time quantitative PCR (qPCR). The primers Q-*SmMYB4*-F and Q-*SmMYB4*-R were synthesized by Beijing Aoke peak Biotechnology Co., Ltd., Beijing, China (Supplementary Material Table S2). *SmUbiquitin* (JF760206.1) served as an internal control to normalize the expression levels. The qPCR reactions were performed in triplicate under the same conditions, as described previously [44]. The relative gene expression was calculated using the comparative C_T_ method.

### 2.5. Determination of the Total Phenolic Acids and Flavonoids

The transgenic and wild *S. miltiorrhiza* were sub-cultured for 90 d. Then, the roots were dried in the shade. The sample preparation followed the methods described by Zhang [45]. The total phenolic acid and flavonoid contents were measured using the Folin–Ciocalteu and NaNO_2_-AlCl_3_ methods, respectively [51,52]. The gallic acid and epicatechin (EC) were used as a standard for determining the total phenolic and flavonoid content, respectively.

### 2.6. Evaluation of the Salvianolic Acid and Tanshinone Contents

The sample preparation followed the previously described method [45]. The contents of the salvianolic acid and tanshinones were determined using a HPLC (high-performance liquid chromatography) system (Agilent Zorbax, Agilent, Santa Clara, CA, USA). The chromatographic separation was performed using a C18 column (Agilent Eclipse XDB-C18-USP L1, 4.6 mm × 250 mm, 5 μm particle size) at 30 °C and a sample injection volume of 20 μL. The detection at 280 nm, using a flow rate of 1.0 mL/min over a gradient of methanol containing 0.4% acetic acid (buffer A), triple distilled water containing 0.4% acetic acid (buffer B), and acetonitrile (Fisher Scientific) containing 0.4% acetic acid (buffer C). The gradient conditions are shown in Supplementary Material Table S3. The standards of rosmarinic acid (RA), salvianolic acid B (SAB), cryptotanshinone (CT), and tanshinone II A (T-II A) were purchased from the National Institute for the Control of Pharmaceutical and Biological Products (Beijing, China).

### 2.7. Expression Analysis of the Essential Genes in the Phenolic Acid and Tanshinone Pathways

Five essential enzyme genes involved in the biosynthesis of salvianolic acids were selected to elucidate the target gene of SmMYB4, including *SmPAL1* (encoding phenylalanine ammonia-lyase, PAL), *SmC4H* (encoding cinnamate-4-hydroxylase, C4H), *Sm4CL2* (encoding 4-coumaric acid CoA-ligase, 4CL), *SmTAT* (encoding tyrosine aminotransferase, TAT), and *SmHPPR* (encoding hydroxyphenylpyruvate reductase, HPPR). Meanwhile, seven key enzyme genes related to the biosynthesis of tanshinones were investigated, including *SmHMGR1-3* (encoding 3-hydroxy-3-methylglutaryl-coenzyme A reductase, HMGR), *SmDXS1*, and *SmDXS3* (encoding 1-deoxy-D-xylulose5-phosphate synthase, DXS), and *SmGGPPS1* and *SmGGPPS3* (encoding geranylgeranyl diphosphate synthase, GGPPS). The sequences of these genes were downloaded from the NCBI database and used to design the qPCR primers (Supplementary Material Table S4).The qPCR was performed according to the method mentioned above.

### 2.8. Analysis of the Target Genes Regulated by SmMYB4

To elucidate the targets of the transcriptional regulation by SmMYB4, the 2.5 kb upstream sequences of the key enzyme genes were extracted from their corresponding scaffolds. The published genome of *S. miltiorrhiza* was obtained from the National Data Center of Traditional Chinese Medicine of China (https://ngdc.cncb.ac.cn/search/?dbId=gwh&q=+PRJCA003150/accessed accessed on 18 August 2020). The amino acid sequence of SmMYB4 was used as the input to the online profile inference tool (http://jaspar.genereg.net/ accessed on 18 August 2020) to search for its binding sequences. The matrix with the smallest E-value was used to scan the promoter sequences of the candidate genes and to ascertain whether they were targets of SmMYB4 (the threshold was set to the default value of 80%).

### 2.9. Yeast One-Hybrid (Y1H) Assay

The Y1H assay was performed, as described in Li et al., 2020 [53].

## 3. Results

### 3.1. Characterization Analysis of SmMYB4

**Figure 1:**
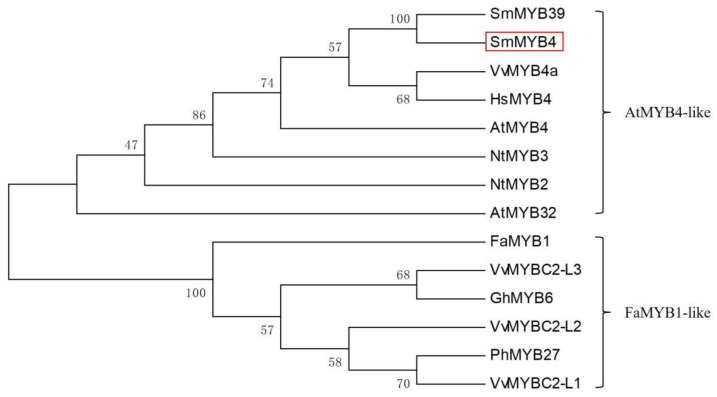
Phylogenetic analysis of the MYB repressors. SmMYB4 is shown by the red rectangle. *S. miltiorrhiza* SmMYB39 (AGS48990.1), SmMYB4 (ADG46002.1), *Arabidopsis thaliana* AtMYB4 (NP_195574.1), AtMYB32 (NP_195225.1), *Fragaria ananassa* FaMYB1 (AAK84064.1), *Narcissus tazetta* NtMYB3 (AGO33166.1), NtMYB2 (ATO58377.1), *Hibiscus syriacus* HsMYB4 (KAE8690675.1), *Petunia hybrid* PhMYB27 (AHX24372.1), *Vitis vinifera* VvMYB4a (NP_001268129.1), VvMYBC2-L1 (ABW34393), VvMYBC2-L2 (ACX50288), VvMYBC2-L3 (AIP98385.1), *Gossypium hirsutum* GhMYB6 (AAN28286.1).

The isolated full-length SmMYB4 contained an ORF comprising 696 bp, encoding a polypeptide of 231 amino acids with a molecular weight of 26 kDa and a pI of 8.865. SmMYB4 is predicted to localize in the nucleus (ProtComp 9.0 server, http://linux1.softberry.com/ accessed on 3 May 2021), and is demonstrated by the further subcellular localization experiment (Supplementary Material Figure S1). The phylogenetic analysis (MEGA6.0) indicated that all R2R3-MYB repressors from various plants are divided into two clades (Figure 1). SmMYB4 is grouped with AtMYB4, AtMYB32, NtMYB2, NtMYB3, and VvMYB4a, belonging to subgroup 4 of the R2R3-MYB transcriptional factors. SmMYB39, which was previously functionally characterized as a repressor of phenolic acid biosynthesis, belongs to the same clade as SmMYB4.

**Figure 2:**
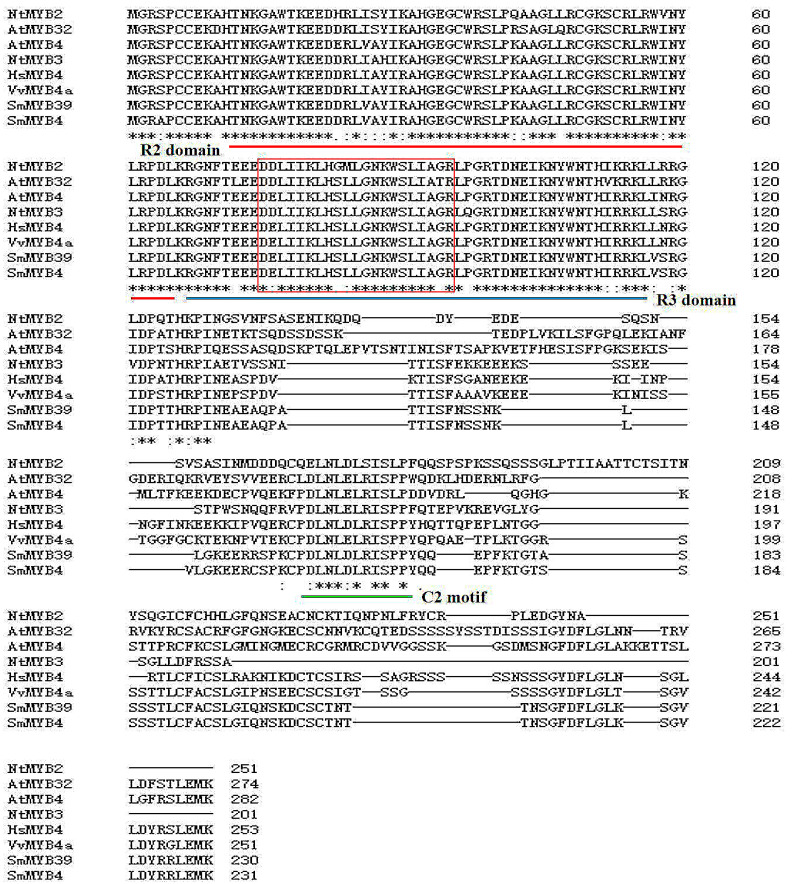
Multiple alignments of the amino acid sequences of SmMYB4 and other R2R3-MYB repressors belonging to subgroup 4. R2 domain, R3 domain, and C2 motif are marked in red, blue, and green lines, respectively. The bHLH binding motif is shown by the red rectangle (*, ** and *** Represents that they have the same conserved structure and function.).

The protein sequence alignment of SmMYB4 and the numerous R2R3-MYB repressors indicated that SmMYB4 consists of conserved R2 and R3 DNA binding domains at the N-terminus. A bHLH binding motif ([D/E]Lx_2_[R/K]x_3_Lx_6_Lx_3_R) was found in the R3 DNA binding domain of SmMYB4. Additionally, the amino acid sequence of SmMYB4 has two typically conserved motifs at the C-terminus, C1 (KLIsrGIDPxT/SHRxI/L) and C2 (pdLNLD/ELxiG/S) (Figure 2), which were previously identified in the subgroup 4 R2R3-MYB transcriptional factors [26].

### 3.2. Molecular Identification of the Transgenic Plantlets

**Figure 3:**
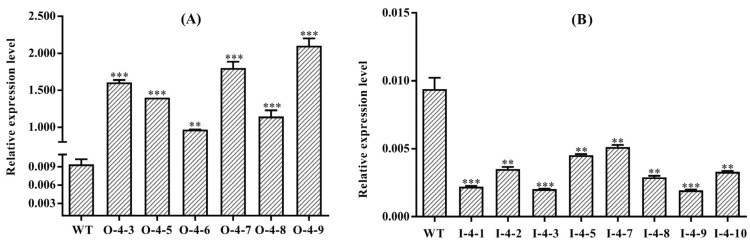
Expression of *SmMYB4* in the transgenic lines. (**A**,B) indicates the expression level of *SmMYB4* in the overexpressing lines and RNAi lines, respectively (** *p* < 0.01, *** *p* < 0.001).

The fragments of about 700 bp were captured from the digestion products of the detected OE*SmMYB4*-1302 vectors, and the expected fragments of approximately 1300 bp and 3400 bp were obtained from the cutting products of the detected IS*mMYB4*-pART27 vectors, which indicated that the overexpression and the RNAi vectors were successfully constructed (Supplementary Material Figure S2). Then, the constructed vectors were sequenced using their respective primers. The transgenic plants were generated to examine the function of SmMYB4. In this research, fourteen overexpression transgenic lines (O-4-1-14) and seventeen interference transgenic lines (I-4-1-16) were screened at the DNA level (Supplementary Material Figure S3). At the level of transcription, six overexpression lines (O-4-3, O-4-5, O-4-6, O-4-7, O-4-8, O-4-9) and eight silent lines (I-4-1, I-4-2, I-4-3, I-4-5, I-4-7, I-4-8, I-4-9, I-4-10) with a significant alteration, were obtained (Figure 3), in which the highest and the lowest expression level of *SmMYB4* was 201.33-fold and 0.207-fold of that in WT, respectively.

### 3.3. SmMYB4 Suppresses the Accumulation of Phenolic Acids and Flavonoids

**Figure 4:**
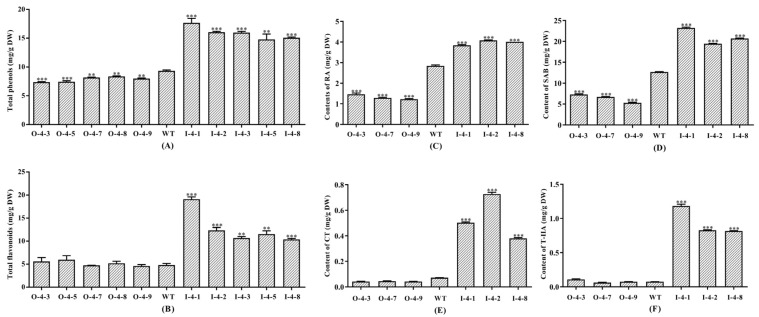
The content of metabolites in the transgenic plants. (**A**–**F**) indicate the content of the total phenolic acids, total flavonoids, RA, SAB, CT, and T-ⅡA, respectively (** *p* < 0.01, *** *p* < 0.001).

To study the function of SmMYB4 in the secondary metabolites biosynthesis, the total phenolic acid and flavonoid contents of *S. miltiorrhiza* were detected (Figure 4A,B). Compared with the WT, the total phenolic acid content was significantly lower in the *SmMYB4* overexpression lines and markedly higher in the RNAi lines. The total phenolic acid content was minimal (7.38 mg/g, DW) in line O-4-3 and maximal (17.67 mg/g, DW) in line I-4-1. The overexpression of SmMYB4 had no significant effect on the flavonoid content, but its silencing resulted in the accumulation of flavonoids, which reached a peak level (19.10 mg/g, DW) in line I-4-1.

### 3.4. SmMYB4 Negatively Regulates the Salvianolic Acid and Tanshinone Biosynthesis

RA and SAB are the most abundant water-soluble active constituents in *S. miltiorrhiza*. It was found that their content had a significant change in the transgenic lines compared with the WT, which had 2.88 mg/g RA and 12.77 mg/g SAB (Figure 4). Compared to the WT, the RA and SAB contents were lower in the overexpression lines (O-4-3, O-4-7, O-4-9). Their respective levels in line O-4-9 notably decreased by 0.566-fold and 0.576-fold. Conversely, their content in the interference lines was significantly higher, being 1.41- and 1.80-fold of their respective contents in the WT (Figure 4C,D).

The total tanshinone content in the overexpression lines was lower than that in the WT, especially in line O-4-9, which was 0.32-fold lower. In contrast, their content in all interference lines was significantly higher, up to 2.98-fold that of WT (Supplementary Material Figure S4). Compared with the WT, the CT content in the three overexpression lines was lower, the lowest being measured in line O-4-9 (0.04 mg/g), whereas the T-IIA content was not significantly changed. It is noteworthy that the content of CT and T-IIA was markedly higher in three interference lines (Figure 4E,F), with line I-4-2 having the most CT (10.14-fold of WT) and line I-4-1 having the most T-IIA (17.2-fold of WT).

### 3.5. SmMYB4 Inhibits the Expression of Key Enzyme Genes

**Figure 5:**
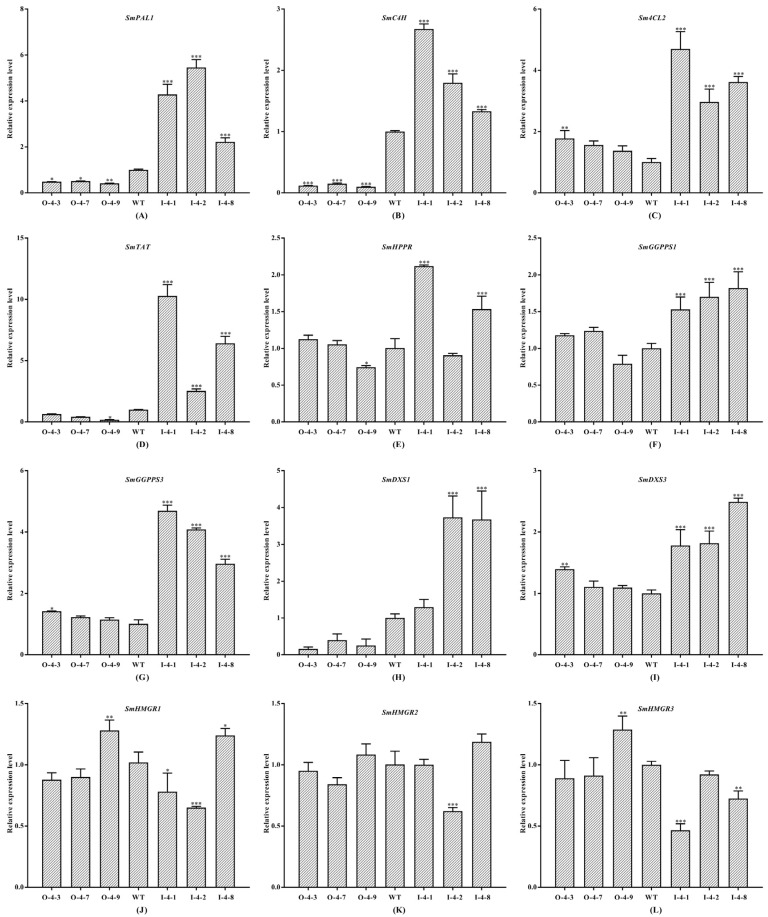
The relative expression level of the key enzyme genes in the salvianolic acid and tanshinone biosynthetic pathways. (**A**–**E**) represent the relative expression level of *SmPAL1*, *SmC4H*, *Sm4CL2*, *SmTAT*, and *SmHPPR* in the salvianolic biosynthetic pathway, respectively. (**F**–**L**) represent the relative expression level of*SmGGPPS1*, *SmGGPPS3*, *SmDXS1*, *SmDXS3*, *SmHMGR1*, *SmHMGR2*, and *SmHMGR3* in the tanshinone biosynthetic pathway, respectively. (* *p* < 0.5, ** *p* < 0.01, *** *p* < 0.001).

To uncover the target genes regulated by SmMYB4, the expression of twelve key enzyme genes involved in the salvianolic acid and tanshinone biosynthetic pathways was analyzed. The qPCR analysis demonstrated that silencing *SmMYB4* markedly upregulated *SmPAL1*, *SmC4H*, *Sm4CL2*, and *SmTAT* (Figure 5). On the contrary, the overexpression of *SmMYB4* downregulated these genes except for *Sm4CL2*. The highest levels of *SmPAL1*, *SmC4H*, *Sm4CL2*, and *SmTAT* were 5.46, 2.67, 4.68, and 10.28 times their respective levels observed in the WT. The lowest levels of *SmPAL1*, *SmC4H*, and *SmTAT* were 41.45%, 9.89%, and 17.02% of their respective levels in the WT. In the tanshinone biosynthetic pathway, *SmDXS1*, *SmDXS3*, *SmGGPPS1*, and *SmGGPPS3* were significantly upregulated in all interference lines, while only *SmDXS1* was downregulated in all overexpressing lines. The transcription levels of other genes increased in the overexpressing lines but were not significantly different from their levels in the WT.

### 3.6. SmMYB4 Possibly Binds to the Promoter of Key Enzyme Genes

**Figure 6:**
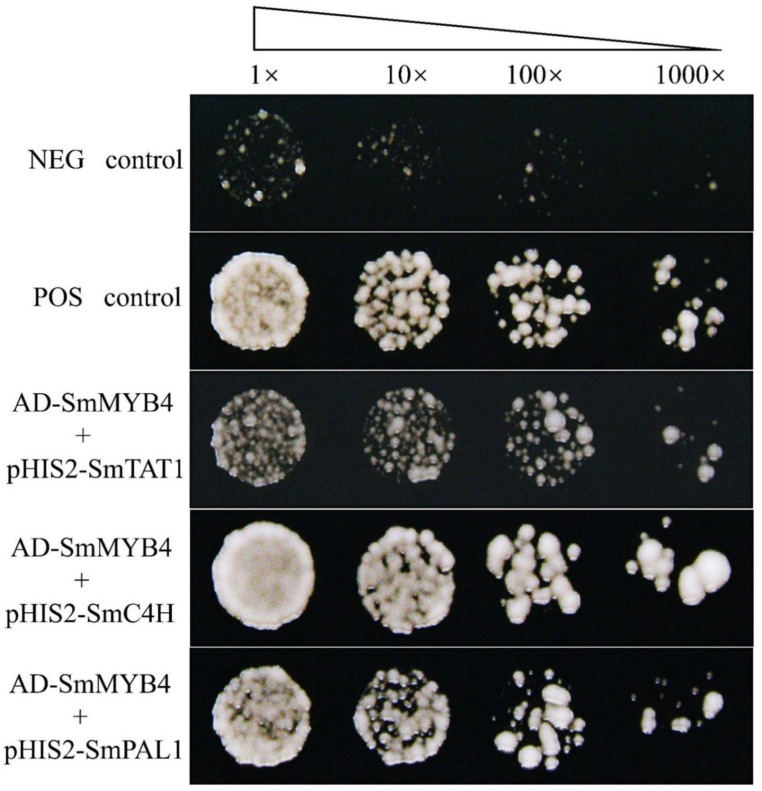
SmMYB4 binds the promoters of SmTAT1, SmC4H, and SmPAL1. The first and second rows represent the negative and positive controls, respectively. NEG control: pGADT7 + p53HIS2; POS control: pGADT7-p53 + p53HIS2.

The matrix {(G/A) (G/T) TG (G/T) T (G/A)} was selected to scan the promoters of *SmPAL1*, *SmC4H*, *Sm4CL2*, *SmTAT*, *SmDXS1*, *SmDXS3*, *SmGGPPS1*, and *SmGGPPS3*. Numerous possible SmMYB4 binding sites were found to exist within each promoter. (Supplementary Material Table S5). The Y1H assay demonstrated that SmMYB4 directly bind the promoters of *SmTAT1*, *SmC4H*, and *SmPAL1* (Figure 6). Therefore, we deduced that SmMYB4 might carry out its inhibitory function in the salvianolic acid and tanshinone biosynthetic pathways by binding to the promoter of these genes.

## 4. Discussion

*S. miltiorrhiza*, an important medicinal material for treating cardiovascular and cerebrovascular diseases, has a growing market demand. The content of the bioactive components in the roots is one of the most crucial quality evaluation indexes. The exclusive information demonstrated that the secondary metabolites biosynthesis is regulated by diverse transcription factors and microRNAs [54,55]. The R2R3-MYB transcriptional factors are a large gene family in plants and have been reported to play crucial roles in regulating secondary metabolites. They can positively and negatively regulate the generation of secondary metabolites.

The R2R3 MYB proteins are more conserved in the N-terminal DNA binding domains, while the protein sequences at the C-terminus are divergent and believed to be responsible for several kinds of regulatory functions. Based on the specific conserved regions in the C-terminus, R2R3 (MYBs TFs) have been classified into many subgroups, in which subgroup 4 R2R3-MYB transcriptional factors function as negative regulators in the phenylpropanoid and flavonoid biosynthesis [56]. Many subgroup 4 R2R3-MYB transcription factors have been identified as transcriptional repressors. AtMYB4 suppressed the expression of *AtC4H* and resulted in the downregulated accumulation of the sinapic acid [57]. The ectopic overexpression of PvMYB4 from *Panicum virgatum* in the transgenic switch grass resulted in a reduced lignin content [58]. BrMYB4 negatively regulated the *BrC4H* gene involved in phenylpropanoid biosynthesis [59]. *NtMYB2* and *NtMYB3* repressed the biosynthesis of anthocyanin and flavonols in the Chinese narcissus, respectively [26,60]. *SmMYB39* and *SmMYB36* negatively regulated the phenolic acid and flavonoid biosynthesis in *S. miltiorrhiza* [45,46]. In the current study, the overexpression of *SmMYB4* significantly decreased the salvianolic acid and tanshinone content, while its silencing enhanced their accumulation, suggesting that *SmMYB4* negatively regulates their biosynthesis. Moreover, a C2 motif that is believed to be the key characteristic responsible for the transcriptional inhibition was harbored in the C terminus of SmMYB4.

**Figure 7:**
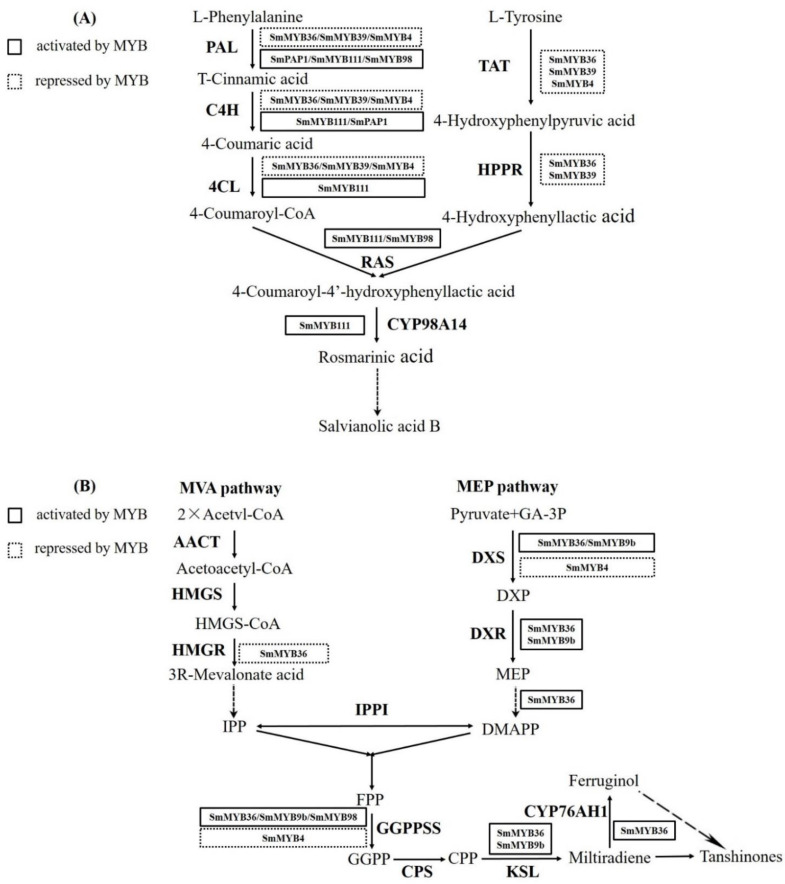
Multiple R2R3-MYBs regulating the salvianolic acid and tanshinone biosynthetic pathways in *S. miltiorrhiza*. (**A**) indicates multiple R2R3-MYBs regulating salvianolic acid synthesis pathway, and (**B**) indicates multiple R2R3-MYBs regulating tanshinone biosynthetic pathway.

The variations in the salvianolic acid and tanshinone content are closely related to the expression of genes encoding key enzymes involved in their biosynthesis. The expression levels of *SmPAL1*, *SmC4H*, *Sm4CL2*, and *SmTAT* were significantly downregulated in the *SmMYB4*-overexpressing transgenic lines and markedly upregulated in the RNAi transgenic lines. Combined with the promoter analysis, it was concluded that SmMYB4 affects the accumulation of the salvianolic acids by binding to the promoters of the genes mentioned above. Nevertheless, SmPAP1 activated the promoters of two key genes, *SmPAL1* and *SmC4H* [43]. *SmMYB98* activated the promoter of *SmPAL1* and *SmRAS1* in the salvianolic acid biosynthesis [47]. The overexpression of *SmMYB36* downregulated most genes in the phenylpropanoid pathway (*SmPAL1*, *SmC4H1*, *Sm4CL2*) and tyrosine pathway (*SmTAT1*, *SmHPPR1*). However, the expression level of *RAS1* and *CYP98A14* did not change significantly. A similar phenomenon occurred in the *SmMYB36*-overexpressing lines [46]. The expression of *SmPAL1*, *SmPAL2*, *SmPAL3*, *SmC4H*, *Sm4CL2*, *Sm4CL3*, *SmRAS1*, and *CYP98A14* was significantly induced in the *SmMYB111*-overexpressing lines but markedly reduced in the corresponding silenced lines [44]. The overexpression of *SmMYB9b* in the hairy roots significantly upregulated the transcription of *SmDXS2*, *SmDXR*, *SmGGPPS*, and *SmKSL1*, suggesting it enhanced the tanshinone synthesis through the stimulation of the MEP (methylerythritol phosphate) pathway [48]. The expression of *SmDXS1*, *SmDXS2*, *SmDXR*, *SmMCT*, *SmMDS*, *SmHDS*, *SmCMK*, *SmHDR1*, *SmHMGR2*, *SmGGPPS1*, *SmCPS1*, *SmKSL1*, and *SmCYP76AHI* was enhanced when *SmMYB36* was overexpressed [46]. *SmMYB98* activated the expression of *SmGGPPS1* in the tanshinone biosynthesis [47]. In our study, the expression level of *SmDXS1*, *SmDXS3 SmGGPPS1*, and *SmGGPPS3* increased significantly in the RNAi transgenic lines but was not changed in the overexpressing lines. We conclude that SmMYB4 affects the accumulation of the tanshinones by negatively regulating the expression of these key enzyme genes. The inability to detect any significant changes in the expression of other genes may be due to the competitive binding of the activator-type MYBs and the repressor-type MYBs to those targets (Figure 7).

The secondary metabolism in plants involves a complex three-dimensional regulatory network. A pathway or a key enzyme gene is usually regulated by many different transcription factors, which can interact and coordinatively regulate the gene expression. For example, PavMYB10.1 interacts with PavbHLH and PavWD40, and binds to the promoter regions of the anthocyanin biosynthesis genes *PavANS* and *PavUFGT* in sweet cherry [61]. The anthocyanin synthesis in the lotus is regulated by a NnMYB5-NnbHLH1-NnTTG1 complex [23]. The MYB activator WHITE PETAL1 (WP1) physically interacts with MtTT8 and MtWD40-1 proteins to form the conserved MYB-basic-helix-loop-helix-WD40 regulatory module, and regulates the floral carotenoid pigmentation through the transcriptional activation of the carotenoid biosynthetic genes in *Medicago truncatula* [29]. SmPAP1 regulates the phenolic acid biosynthetic pathway in *S. miltiorrhiza* by interacting with SmMYC2 and activating *SmPAL1* and *SmC4H* [43]. SmMYB111 positively regulates the phenolic acid biosynthesis in *S. miltiorrhiza* by interacting with SmTTG1 and SmbHLH51 [44]. However, the specific mechanism of SmMYB4 needs further study. Our work will entail not only the elucidation of the secondary metabolic networks, but also enhance the understanding of the gene regulatory networks integrated with the metabolic networks.

## 5. Conclusions

In summary, a subgroup 4 R2R3 MYB transcription factor gene named *SmMYB4* was cloned and functionally identified in the present study. Six overexpressions and eight RNA interference transgenic lines were successfully obtained via the *Agrobacterium*-mediated leaf-disk transformed method. Based on the analysis of the content of the phenolic acids and tanshinones in transgenic plants and the expression of genes in the related pathway, we speculated that SmMYB4 negatively regulates the biosynthesis of the phenolic acids by repressing the expression of *SmPAL1*, *SmC4H*, *Sm4CL2*, and *SmTAT*, and simultaneously negatively regulates the biosynthesis of tanshinones by inhibiting the expression of *SmDXS1*, *SmDXS3 SmGGPPS1*, and *SmGGPPS3*. SmMYB4 may directly bind to the promoter of these genes. Our results will be helpful in better understanding the regulation mechanism of the phenolic acids and tanshinones production in *S. miltiorrhiza* and provide a train of thought to improve the contents of bioactive compounds in this traditional herbal.

## Data Availability

The data presented in this study are available in the article and supplementary materials.

## Supplementary Materials

The following supporting information can be downloaded at: https://www.mdpi.com/article/10.3390/metabo12100968/s1, Table S1: Primers used in the *SmMYB4*-OE and *SmMYB4*-RNAi vector construction; Table S2: Primers for the molecular detection of transgenic plants; Table S3: Gradient conditions of the mobile phase of HPLC; Table S4: Primers for the key enzyme genes in the salvianolic acid and tanshinone biosynthetic pathways; Table S5: Information of the SmMYB4 binding sites in the promoters of the key enzyme genes; Figure S1: Subcellular localization of SmMYB4 in onion epidermal cells; Figure S2: Restriction endonuclease digestion of the overexpression and RNAi expression vectors. A represents the Bgl II/BstP I digestion of the overexpression vectors, in which P represents plasmids, M refers to Marker DL2000, and 1–4 represents the digest results of Bgl II/BstP I. B represents the digestion of the RNAi expression vectors, in which P represents plasmids, M refers to Marker DL2000, 1 refers to the digestion of Xho I, and 2 refers to the digestion of Not I. The target fragment is marked with a red line or arrows; Figure S3: Detection of the transgenic lines at the DNA level Detection. Results of the overexpression and interference transgenic lines were individually shown in Figure S3A,B: P indicates positive control (Plasmids), WT indicates negative control (untransformed plants), and M indicates Marker DL2000; Figure S4: Total tanshinone contents in the roots of transgenic plants (** *p* < 0.01, *** *p* < 0.001).

## References

[1] Ren J., Fu L., Nile S.H., Zhang J., Kai G. (2019). *Salvia miltiorrhiza* in treating cardiovascular diseases: A review on its pharmacological and clinical applications. Front. Pharmacol..

[2] Gantet P., Memelink J. (2002). Transcription factors: Tools to engineer the production of pharmacologically active plant metabolites. Trends Pharmacol. Sci..

[3] Xu H., Song J., Luo H., Zhang Y., Li Q., Zhu Y., Xu J., Li Y., Song C., Wang B. (2016). Analysis of the genome sequence of the medicinal plant *Salvia miltiorrhiza*. Mol. Plant.

[4] Li C., Lu S. (2014). Genome-wide characterization and comparative analysis of R2R3-MYB transcription factors shows the complexity of MYB-associated regulatory networks in *Salvia miltiorrhiza*. BMC Genom..

[5] Zhang X., Luo H., Xu Z., Zhu Y., Ji A., Song J., Chen S. (2015). Genome-wide characterisation and analysis of bHLH transcription factors related to tanshinone biosynthesis in *Salvia miltiorrhiza*. Sci. Rep..

[6] Yu H., Guo W., Yang D., Hou Z., Liang Z. (2018). Transcriptional profiles of *SmWRKY* family genes and their putative roles in the biosynthesis of tanshinone and phenolic acids in *Salvia miltiorrhiza*. Int. J. Mol. Sci..

[7] Ji A.J., Luo H.M., Xu Z.C., Zhang X., Zhu Y.J., Liao B.S., Yao H., Song J.Y., Chen S.L. (2016). Genome-wide identification of the AP2/ERF gene family involved in active constituent biosynthesis in *Salvia miltiorrhiza*. Plant Genome.

[8] Zhang Y., Xu Z., Ji A., Luo H., Song J. (2018). Genomic survey of bZIP transcription factor genes related to tanshinone biosynthesis in *Salvia miltiorrhiza*. Acta Pharm. Sin. B.

[9] Xing B., Yang D., Yu H., Zhang B., Yan K., Zhang X., Han R., Liang Z. (2018). Overexpression of *SmbHLH10* enhances tanshinones biosynthesis in *Salvia miltiorrhiza* hairy roots. Plant Sci..

[10] Cao W., Wang Y., Shi M., Hao X., Zhao W., Wang Y., Ren J., Kai G. (2018). Transcription factor SmWRKY1 positively promotes the biosynthesis of tanshinones in *Salvia miltiorrhiza*. Front Plant Sci..

[11] Deng C., Hao X., Shi M., Fu R., Wang Y., Zhang Y., Zhou W., Feng Y., Makunga N.P., Kai G. (2019). Tanshinone production could be increased by the expression of *SmWRKY2* in *Salvia miltiorrhiza* hairy roots. Plant Sci..

[12] Wu Y., Zhang Y., Li L., Guo X., Wang B., Cao X., Wang Z. (2018). AtPAP1 interacts with and activates SmbHLH51, a positive regulator to phenolic acids biosynthesis in *Salvia miltiorrhiza*. Front Plant Sci..

[13] Xing B., Liang L., Liu L., Hou Z., Yang D., Yan K., Zhang X., Liang Z. (2018). Overexpression of *SmbHLH148* induced biosynthesis of tanshinones as well as phenolic acids in *Salvia miltiorrhiza* hairy roots. Plant Cell Rep..

[14] Zhang C., Xing B., Yang D., Ren M., Guo H., Yang S., Liang Z. (2020). SmbHLH3 acts as a transcription repressor for both phenolic acids and tanshinone biosynthesis in *Salvia miltiorrhiza* hairy roots. Phytochemistry.

[15] Sun M., Shi M., Wang Y., Huang Q., Yuan T., Wang Q., Wang C., Zhou W., Kai G. (2019). The biosynthesis of phenolic acids is positively regulated by the JA-responsive transcription factor ERF115 in *Salvia miltiorrhiza*. J. Exp. Bot..

[16] Li W., Bai Z., Pei T., Yang D., Mao R., Zhang B., Liu C., Liang Z. (2019). SmGRAS1 and SmGRAS2 regulate the biosynthesis of tanshinones and phenolic acids in *Salvia miltiorrhiza*. Front Plant Sci..

[17] Zhou Y., Zhu H., He S., Zhai H., Zhao N., Xing S., Wei Z., Liu Q. (2019). A novel sweetpotato transcription factor gene *IbMYB116* enhances drought tolerance in transgenic *Arabidopsis*. Front Plant Sci..

[18] Dong W., Liu X., Li D., Gao T., Song Y. (2018). Transcriptional profiling reveals that a MYB transcription factor *MsMYB4* contributes to the salinity stress response of alfalfa. PLoS ONE.

[19] Xing C., Liu Y., Zhao L., Zhang S., Huang X. (2019). A novel MYB transcription factor regulates ascorbic acid synthesis and affects cold tolerance. Plant Cell Environ..

[20] Schwinn K.E., Ngo H., Kenel F., Brummell D.A., Albert N.W., McCallum J.A., Pither-Joyce M., Crowhurst R.N., Eady C., Davies K.M. (2016). The onion (*Allium cepa* L.) *R2R3-MYB* gene *MYB1* regulates anthocyanin biosynthesis. Front Plant Sci..

[21] Yu M., Man Y., Wang Y. (2019). Light- and temperature-induced expression of an R2R3-MYB gene regulates anthocyanin biosynthesis in red-fleshed kiwifruit. Int. J. Mol. Sci..

[22] Oglesby L., Ananga A., Obuya J., Ochieng J., Cebert E., Tsolova V. (2016). Anthocyanin accumulation in muscadine berry skins is influenced by the expression of the MYB transcription factors, *MybA1*, and *MYBCS1*. Antioxidants.

[23] Sun S.S., Gugger P.F., Wang Q.F., Chen J.M. (2016). Identification of a *R2R3-MYB* gene regulating anthocyanin biosynthesis and relationships between its variation and flower color difference in lotus (*Nelumbo* Adans.). PeerJ.

[24] Blanco E., Sabetta W., Danzi D., Negro D., Passeri V., Lisi A., Paolocci F., Sonnante G. (2018). Isolation and characterization of the flavonol regulator CcMYB12 from the globe artichoke [*Cynara cardunculus* var. scolymus (L.) Fiori]. Front Plant Sci..

[25] Matsui K., Oshima Y., Mitsuda N., Sakamoto S., Nishiba Y., Walker A.R., Ohme-Takagi M., Robinson S.P., Yasui Y., Mori M. (2018). Buckwheat R2R3 MYB transcription factor FeMYBF1 regulates flavonol biosynthesis. Plant Sci..

[26] Anwar M., Yu W., Yao H., Zhou P., Allan A.C., Zeng L. (2019). *NtMYB3*, an R2R3-MYB from Narcissus, regulates flavonoid biosynthesis. Int. J. Mol. Sci..

[27] An C., Sheng L., Du X., Wang Y., Zhang Y., Song A., Jiang J., Guan Z., Fang W., Chen F. (2019). Overexpression of *CmMYB15* provides chrysanthemum resistance to aphids by regulating the biosynthesis of lignin. Hortic. Res..

[28] Ampomah-Dwamena C., Thrimawithana A.H., Dejnoprat S., Lewis D., Espley R.V., Allan A.C. (2019). A kiwifruit (*Actinidia deliciosa*) R2R3-MYB transcription factor modulates chlorophyll and carotenoid accumulation. New Phytol..

[29] Meng Y., Wang Z., Wang Y., Wang C., Zhu B., Liu H., Ji W., Wen J., Chu C., Tadege M. (2019). The MYB activator WHITE PETAL1 associates with MtTT8 and MtWD40-1 to regulate carotenoid-derived flower pigmentation in *Medicago truncatula*. Plant Cell.

[30] Ge L., Dou Y., Li M., Qu P., He Z., Liu Y., Xu Z., Chen J., Chen M., Ma Y. (2019). SiMYB3 in foxtail millet (*Setaria italica*) confers tolerance to low-nitrogen stress by regulating root growth in transgenic plants. Int. J. Mol. Sci..

[31] Yang Y., Zhang L., Chen P., Liang T., Li X., Liu H. (2020). UV-B photoreceptor UVR8 interacts with MYB73/MYB77 to regulate auxin responses and lateral root development. EMBO J..

[32] Zhang L., Dong C., Zhang Q., Zhao G., Li F., Xia C., Zhang L., Han L., Wu J., Jia J. (2016). The wheat MYB transcription factor TaMYB18 regulates leaf rolling in rice. Biochem. Biophys. Res. Commun..

[33] Fan Z.Q., Ba L.J., Shan W., Xiao Y.Y., Lu W.J., Kuang J.F., Chen J.Y. (2018). A banana R2R3-MYB transcription factor MaMYB3 is involved in fruit ripening through modulation of starch degradation by repressing starch degradation-related genes and *MabHLH6*. Plant J..

[34] Wang F.P., Wang X.F., Zhang J., Ma F., Hao Y.J. (2018). MdMYB58 modulates Fe homeostasis by directly binding to the *MdMATE43* promoter in plants. Plant Cell Physiol..

[35] Yu Y., Guo D., Li G., Yang Y., Zhang G., Li S., Liang Z. (2019). The grapevine R2R3-type MYB transcription factor VdMYB1 positively regulates defense responses by activating the *stilbene synthase gene 2* (*VdSTS2*). BMC Plant Biol..

[36] Zhang Y.L., Zhang C.L., Wang G.L., Wang Y.X., Qi C.H., Zhao Q., You C.X., Li Y.Y., Hao Y.J. (2019). The R2R3 MYB transcription factor MdMYB30 modulates plant resistance against pathogens by regulating cuticular wax biosynthesis. BMC Plant Biol..

[37] Qiu Z., Yan S., Xia B., Jiang J., Yu B., Lei J., Chen C., Chen L., Yang Y., Wang Y. (2019). The eggplant transcription factor MYB44 enhances resistance to bacterial wilt by activating the expression of *spermidine synthase*. J. Exp. Bot..

[38] Li Z., Peng R., Tian Y., Han H., Xu J., Yao Q. (2016). Genome-wide identification and analysis of the MYB transcription factor superfamily in *Solanum lycopersicum*. Plant Cell Physiol..

[39] Salih H., Gong W., He S., Sun G., Sun J., Du X. (2016). Genome-wide characterization and expression analysis of MYB transcription factors in *Gossypium hirsutum*. BMC Genet.

[40] Zhang C., Ma R., Xu J., Yan J., Guo L., Song J., Feng R., Yu M. (2018). Genome-wide identification and classification of *MYB* superfamily genes in peach. PLoS ONE.

[41] Sun W., Ma Z., Chen H., Liu M. (2019). MYB gene family in potato (*Solanum tuberosum* L.): Genome-wide identification of hormone-responsive reveals their potential functions in growth and development. Int. J. Mol. Sci..

[42] Zhou Q., Jia C., Ma W., Cui Y., Jin X., Luo D., Min X., Liu Z. (2019). MYB transcription factors in alfalfa (*Medicago sativa*): Genome-wide identification and expression analysis under abiotic stresses. PeerJ.

[43] Hao G., Jiang X., Feng L., Tao R., Li Y., Huang L. (2015). Cloning, molecular characterization and functional analysis of a putative R2R3-MYB transcription factor of the phenolic acid biosynthetic pathway in *S. miltiorrhiza* Bge. f. alba. Plant Cell Tissue Organ. Cult. (PCTOC).

[44] Li S., Wu Y., Kuang J., Wang H., Du T., Huang Y., Zhang Y., Cao X., Wang Z. (2018). SmMYB111 is a key factor to phenolic acid biosynthesis and interacts with both SmTTG1 and SmbHLH51 in *Salvia miltiorrhiza*. J. Agric. Food Chem..

[45] Zhang S., Ma P., Yang D., Li W., Liang Z., Liu Y., Liu F. (2013). Cloning and characterization of a putative R2R3 MYB transcriptional repressor of the rosmarinic acid biosynthetic pathway from *Salvia miltiorrhiza*. PLoS ONE.

[46] Ding K., Pei T., Bai Z., Jia Y., Ma P., Liang Z. (2017). SmMYB36, a novel R2R3-MYB transcription factor, enhances tanshinone accumulation and decreases phenolic acid content in *Salvia miltiorrhiza* hairy roots. Sci. Rep..

[47] Hao X., Pu Z., Cao G., You D., Zhou Y., Deng C., Shi M., Nile S.H., Wang Y., Zhou W. (2020). Tanshinone and salvianolic acid biosynthesis are regulated by *SmMYB98* in *Salvia miltiorrhiza* hairy roots. J. Adv. Res..

[48] Zhang J., Zhou L., Zheng X., Zhang J., Yang L., Tan R., Zhao S. (2017). Overexpression of *SmMYB9b* enhances tanshinone concentration in *Salvia miltiorrhiza* hairy roots. Plant Cell Rep..

[49] Yan Y.-P., Wang Z.-Z. (2007). Genetic transformation of the medicinal plant *Salvia miltiorrhiza* by *Agrobacterium tumefaciens*-mediated method. Plant Cell Tissue Organ Cult..

[50] McCormac A.C., Elliott M.C., Chen D.F. (1998). A simple method for the production of highly competent cells of *Agrobacterium* for transformation via electroporation. Mol. Biotechnol..

[51] Yan Q., Shi M., Ng J., Wu J.Y. (2006). Elicitor-induced rosmarinic acid accumulation and secondary metabolism enzyme activities in *Salvia miltiorrhiza* hairy roots. Plant Sci..

[52] Dewanto V., Wu X., Adom K.K., Liu R.H. (2002). Thermal processing enhances the nutritional value of tomatoes by increasing total antioxidant activity. J. Agric. Food Chem..

[53] Li L., Wang D., Zhou L., Yu X., Yan X., Zhang Q., Li B., Liu Y., Zhou W., Cao X. (2020). JA-responsive transcription factor SmMYB97 promotes phenolic acid and tanshinone accumulation in *Salvia miltiorrhiza*. J. Agric. Food Chem..

[54] Bai Z., Wu J., Huang W., Jiao J., Zhang C., Hou Z., Yan K., Zhang X., Han R., Liang Z. (2020). The ethylene response factor *SmERF8* regulates the expression of *SmKSL1* and is involved in tanshinone biosynthesis in *Saliva miltiorrhiza* hairy roots. J. Plant Physiol..

[55] Xu X., Jiang Q., Ma X., Ying Q., Shen B., Qian Y., Song H., Wang H. (2014). Deep sequencing identifies tissue-specific microRNAs and their target genes involving in the biosynthesis of tanshinones in *Salvia miltiorrhiza*. PLoS ONE.

[56] Zhou M., Zhang K., Sun Z., Yan M., Chen C., Zhang X., Tang Y., Wu Y. (2017). LNK1 and LNK2 corepressors interact with the MYB3 transcription factor in phenylpropanoid biosynthesis. Plant Physiol..

[57] Jin H., Cominelli E., Bailey P., Parr A., Mehrtens F., Jones J., Tonelli C., Weisshaar B., Martin C. (2000). Transcriptional repression by AtMYB4 controls production of UV-protecting sunscreens in *Arabidopsis*. Embo J..

[58] Shen H., He X., Poovaiah C.R., Wuddineh W.A., Ma J., Mann D.G.J., Wang H., Jackson L., Tang Y., Neal Stewart C. (2012). Functional characterization of the switchgrass (*Panicum virgatum*) R2R3-MYB transcription factor *PvMYB4* for improvement of lignocellulosic feedstocks. New Phytol..

[59] Zhang L., Wang Y., Sun M., Wang J., Kawabata S., Li Y. (2014). *BrMYB4*, a suppressor of genes for phenylpropanoid and anthocyanin biosynthesis, is down-regulated by UV-B but not by pigment-inducing sunlight in turnip cv. Tsuda. Plant Cell Physiol..

[60] Anwar M., Wang G., Wu J., Waheed S., Allan A.C., Zeng L. (2018). Ectopic overexpression of a novel R2R3-MYB, *NtMYB2* from Chinese narcissus represses anthocyanin biosynthesis in tobacco. Molecules.

[61] Jin W., Wang H., Li M., Wang J., Yang Y., Zhang X., Yan G., Zhang H., Liu J., Zhang K. (2016). The R2R3 MYB transcription factor *PavMYB10.1* involves in anthocyanin biosynthesis and determines fruit skin colour in sweet cherry (*Prunus avium* L.). Plant Biotechnol. J..

