# Supplementary material for: SmMYB4 Is a R2R3-MYB Transcriptional Repressor Regulating the Biosynthesis of Phenolic Acids and Tanshinones in Salvia miltiorrhiza
Source: Metabolites. 2022 Oct 12;12(10):968. doi: 10.3390/metabo12100968 (PMC9609640; doi:10.3390/metabo12100968)
Supplement: Supplementary file 1 [file metabolites-12-00968-s001.zip › metabolites-1932614-supplementary.pdf]

Table S1. Primers used in the *SmMYB4*-OE and *SmMYB4*-RNAi vector construction.

Primer	Sequence(5′-3′)
OESmMYB4-Bgl II	5′-GAAGATCTTCATGGGAAGGGCTCCATGCT-3′
*OESmMYB4-Bst*P I	5′-GGGTAACCCCTTCATTTCATCTCCAATCTTCTGT-3′
*ISmMYB4-Hin*d III	5′-AAGCTTAGCTGGTGAGCCGAGGCATT-3′
ISmMYB4-Kpn I	5′-GGTACCAGCTGGTGAGCCGAGGCATT-3′
*ISmMYB4-Bam*H I	5′-GGATCCTGCCGAGACTACAAGCGAAGC-3′
ISmMYB4-Xho I	5′-CTCGAGTGCCGAGACTACAAGCGAAGC-3′

Table S2. Primers for the molecular detection of transgenic plants.

Name of primers	Sequence of primers (5′-3′)
1302-*OESmMYB4*-F	5′-ATAGAGGACCTAACAGAACTCGCCG-3′
1302-*OESmMYB4*-R	5′-GCCGATCGTCTTCTTCCTTAGTCC-3′
In-*ISmMYB4*-F	5′-GACGCACAATCCCACTATCCTT-3′
In-*ISmMYB4*-R	5′-AGACTACAAGCGAAGCACAAGGT-3′
Q-*MYB4*-F	5′-GGCTCAGCCTGCCACAACCA-3′
Q-*MYB4*-R	5′-GTACCTGTTTTGAAGGGTTCTTGTTGA-3′
SmUbiquitin-F	5′-ACCCTCACGGGGAAGACCATC-3′
SmUbiquitin-R	5′-ACCACGGAGACGGAGGACAAG-3′

Table S3. Gradient conditions of the mobile phase of HPLC.

Time (min)	A (%)	B (%)	C (%)
0.00	0.00	95.00	5.00
5.00	0.00	90.00	10.00
25.00	3.00	67.00	30.00
40.00	5.00	60.00	35.00
60.00	10.00	35.00	55.00
70.00	15.00	25.00	60.00
75.00	20.00	10.00	70.00
80.00	20.00	0.00	80.00

Table S4. Primers for the key enzyme genes in the salvianolic acid and tanshinone biosynthetic pathways.

Primers	Sequence (5′-3′)
SmPAL1-F	5′-GGCGGCGATTGAGAGCAGGA-3′
SmPAL1-R	5′-ATCAGCAGATAGGAAGAGGAGCACC-3′
SmC4H-F	5′-GGCGGCGATTGAGAGCAGGA-3′
SmC4H-R	5′-GCCACCAAGCGTTCACCAAGAT-3′
Sm4CL2-F	5′- TCGCCAAATACGACCTTTCC-3′
Sm4CL2-R	5′-TGCTTCAGTCATCCCATACCC-3′
SmTAT-F	5′-CAACTGCTGGTCTTCCACAAAC-3′
SmTAT-R	5′-GCGAGCCAAAACGGACA-3’
SmHPPR-F	5′-TGACTCCAGAAACAACCCACATT-3′
SmHPPR-R	5′-CCCAGACGACCCTCCACAAG-3′
SmHMGR1-F	5'- GCAACCATCTACTCTCGTCCCA-3'
SmHMGR1-R	5'- GTGCTCCATGAGCTGCATCAG-3'
SmHMGR2-F	5'-GGGTTCAACTACGAGGCCATACTG-3'
SmHMGR2-R	5'-TGTTTGTGCTCGCCACCAGG-3'
SmHMGR3-F	5′-AGTCTCGTGATGTCCCTGCTCG-3′
SmHMGR3-R	5′- GCCTCAACCTGCTTGGCGTA-3′
SmDXS1-F	5'-TGAGAGCGACTACGACTGCTTTGG-3'
SmDXS1-R	5'-CCCATCCAGATTGGCAGTAGGC-3'
SmDXS3-F	5′-CACGAATGGGCTGCCAAAAT-3′
SmDXS3-R	5′-CCATCGAATCCAATGAAGCCAC-3′
SmGGPPS1-F	5'-GGGGCTATTTTGGGAGGTGGAA-3'
SmGGPPS1-R	5'-CAGCAGCTTGGGATACGTGGTC-3'
SmGGPPS3-F	5′-GGCCAGTGCTCTGCTGTCTGTG-3′
SmGGPPS3-R	5′-TCGGCCACCTCCATCGCTT-3′

Table S5. Information of the SmMYB4 binding sites in the promoters of the key enzyme genes.

Promoter of genes	Matrix ID	Score	Relative score	Start	End	Strand	Sequence
P-*SmPAL1*	MA1039.1	12.6546	1.00000000264	1153	1160	-	GGTAGGTG
	MA1039.1	12.1323	0.988195208085	982	989	-	GGTTGGTG
	MA1039.1	8.39756	0.903775442929	1107	1114	-	GTTTGTTG
	MA1039.1	6.91318	0.870222818545	985	992	-	GGTGGTTG
	MA1039.1	6.79929	0.867648440449	947	954	-	ATTAGTTA
	MA1039.1	6.44284	0.859591426411	6	13	+	TTTTGGTG
	MA1039.1	5.89032	0.847102392325	316	323	+	AGTTAGTG
	MA1039.1	5.70836	0.842989388734	104	111	+	TTTTGGTA
	MA1039.1	5.70836	0.842989388734	1157	1164	-	TTTTGGTA
	MA1039.1	5.67809	0.842305159977	904	911	+	AGTAAGTA
	MA1039.1	5.42622	0.836611851162	1294	1301	-	CGTTGGTT
	MA1039.1	5.33172	0.834475956326	1220	1227	-	GTTAAGTA
	MA1039.1	5.22975	0.832171014456	1227	1234	-	AATTGGTG
	MA1039.1	5.17587	0.830953086836	389	396	+	GGTTTGTG
	MA1039.1	5.15111	0.830393326017	1235	1242	-	GGATGGTG
	MA1039.1	5.1218	0.829730783222	458	465	+	GTTAGGAG
	MA1039.1	4.53953	0.816569322629	854	861	+	GGTTATTA
	MA1039.1	4.09273	0.806469958718	474	481	+	ATTTGATG
P-*SmC4H*	MA1039.1	12.6546	1.00000000264	1453	1460	-	GGTAGGTG
	MA1039.1	12.6546	1.00000000264	1771	1778	-	GGTAGGTG
	MA1039.1	12.1323	0.988195208085	150	157	+	GGTTGGTG
	MA1039.1	10.7463	0.956865437953	1950	1957	-	AGTTGGTG
	MA1039.1	10.3999	0.949036223524	1759	1766	-	GTTTGGTG
	MA1039.1	9.01388	0.917706474949	752	759	-	ATTTGGTG
	MA1039.1	8.2794	0.90110444805	1591	1598	-	ATTTGGTA
	MA1039.1	7.79862	0.890236957007	1800	1807	-	GGTAAGTG
	MA1039.1	6.96509	0.871396220961	1882	1889	-	TTTAGGTG
	MA1039.1	6.91318	0.870222818545	147	154	+	GGTGGTTG
	MA1039.1	6.79929	0.867648440449	622	629	+	ATTAGTTA
	MA1039.1	6.1665	0.853345082444	72	79	+	GGTGGGTT
	MA1039.1	5.67336	0.842198131346	1867	1874	-	GGGAGGTG
	MA1039.1	5.15255	0.830425995079	1912	1919	-	GGTTGGGG
	MA1039.1	5.15111	0.830393326017	102	109	+	GGGTGGTG
	MA1039.1	5.15111	0.830393326017	1809	1816	-	GGATGGTG
	MA1039.1	5.09066	0.829026894824	500	507	+	AGTTGATA
	MA1039.1	4.94591	0.825755192322	555	562	-	AGTTGGAG
	MA1039.1	4.68016	0.819748223871	1086	1093	-	ATTAAGTG

	MA1039.1	4.68016	0.819748223871	1317	1324	-	ATTAAGTG
	MA1039.1	4.59955	0.817925988672	444	451	-	GTTTGGAG
	MA1039.1	4.41807	0.813823957402	133	140	+	GGTTGGGA
	MA1039.1	4.16415	0.808084280293	1946	1953	-	GGTGGTTT
	MA1039.1	4.09273	0.806469958718	140	147	+	ATTTGATG
	MA1039.1	3.99439	0.804247104467	12	19	+	GGTGGCTG
	MA1039.1	3.99439	0.804247104467	261	268	+	GGTGGCTG
	MA1039.1	3.94568	0.803146191583	1333	1340	+	ATTAAGTA
	MA1039.1	3.8805	0.80167272637	1075	1082	+	ATTAGATA
P-*Sm4CL2*	MA1039.1	12.1323	0.988195208085	1978	1985	-	GGTTGGTG
	MA1039.1	10.7463	0.956865437953	2047	2054	-	AGTTGGTG
	MA1039.1	10.7463	0.956865437953	2066	2073	-	AGTTGGTG
	MA1039.1	10.5341	0.952068205605	1867	1874	+	AGTAGGTA
	MA1039.1	7.44077	0.882148362517	1560	1567	-	TGTTGGTA
	MA1039.1	7.38094	0.880795867681	2030	2037	-	GGTTGTTT
	MA1039.1	7.21118	0.876958681076	2080	2087	-	GGTTGATG
	MA1039.1	6.85421	0.868889757004	2072	2079	-	GGTAGGAG
	MA1039.1	6.51714	0.861270902877	4	11	-	AGTAGTTC
	MA1039.1	6.26484	0.855567936696	1442	1449	-	ATTTGGTC
	MA1039.1	6.17078	0.853441699227	1252	1259	-	GTTAGTTC
	MA1039.1	5.42622	0.836611851162	624	631	-	CGTTGGTT
	MA1039.1	5.26654	0.833002485724	1395	1402	+	GTTAGATA
	MA1039.1	5.26654	0.833002485724	1839	1846	-	GTTAGATA
	MA1039.1	4.95846	0.826038812805	1964	1971	-	CGTGGGT
	MA1039.1	4.46214	0.814820142824	189	196	-	GGTTGATC
	MA1039.1	4.46214	0.814820142824	907	914	-	GGTTGATC
	MA1039.1	4.44049	0.814330624259	1563	1570	-	TTTTGTTG
	MA1039.1	4.38901	0.813167030107	1501	1508	-	GATAGGTT
	MA1039.1	4.21606	0.809257682708	2122	2129	+	TTTAGGTT
	MA1039.1	4.11737	0.807026927954	1686	1693	+	GGTAGTAA
	MA1039.1	4.02082	0.804844546262	911	918	-	GGCAGGTT
P-*SmTAT*	MA1039.1	12.6546	1.00000000264	1724	1731	-	GGTAGGTG
	MA1039.1	10.3999	0.949036223524	1932	1939	-	GTTTGGTG
	MA1039.1	8.80165	0.912909242601	1743	1750	-	ATTAGGTA
	MA1039.1	7.66308	0.887173405253	664	671	+	GTTTGTTA
	MA1039.1	7.66308	0.887173405253	1262	1269	+	GTTTGTTA
	MA1039.1	6.79929	0.867648440449	2139	2146	-	ATTAGTTA
	MA1039.1	6.78709	0.867372731246	1564	1571	+	ATTAGGTT
	MA1039.1	6.69515	0.865294392592	95	102	+	TGTAGTTG
	MA1039.1	6.69515	0.865294392592	987	994	+	TGTAGTTG
	MA1039.1	6.69515	0.865294392592	1589	1596	+	TGTAGTTG
	MA1039.1	6.26484	0.855567936696	296	303	-	ATTTGGTC

	MA1039.1	6.26484	0.855567936696	369	376	-	ATTTGGTC
	MA1039.1	5.64853	0.841636893898	2387	2394	-	GTTTGTTT
	MA1039.1	4.41022	0.813646395502	309	316	+	AGTAATTG
	MA1039.1	4.41022	0.813646395502	382	389	+	AGTAATTG
	MA1039.1	4.26249	0.810307134544	861	868	-	ATTTGTTT
	MA1039.1	4.26249	0.810307134544	1459	1466	-	ATTTGTTT
	MA1039.1	4.21606	0.809257682708	1956	1963	-	TTTAGGTC
	MA1039.1	3.94611	0.803155854339	677	684	+	TGTAGTTT
	MA1039.1	3.94611	0.803155854339	892	899	+	TGTAGTTT
	MA1039.1	3.94611	0.803155854339	1275	1282	+	TGTAGTTT
	MA1039.1	3.94611	0.803155854339	1490	1497	+	TGTAGTTT
	MA1039.1	3.88797	0.801841600952	848	855	+	AGTTATTG
	MA1039.1	3.88797	0.801841600952	1446	1453	+	AGTTATTG
P-*SmDXS1*	MA1039.1	9.26618	0.923409451908	1996	2003	-	AGTAGTTG
	MA1039.1	8.6975	0.910555194744	1201	1208	+	TGTAGGTG
	MA1039.1	8.17525	0.898750400194	2276	2283	-	TGTTGGTG
	MA1039.1	7.99725	0.8947268997	530	537	-	AGTTGGTT
	MA1039.1	7.65089	0.88689769605	741	748	+	GTTTGGTT
	MA1039.1	7.65089	0.88689769605	1017	1024	-	GTTTGGTT
	MA1039.1	7.53377	0.884250478126	631	638	+	ATTAGTTG
	MA1039.1	7.38094	0.880795867681	1021	1028	-	GGTTGTTT
	MA1039.1	7.18313	0.876324646914	746	753	+	GTTGGGTG
	MA1039.1	7.01152	0.872445672797	1359	1366	+	ATTTGTTG
	MA1039.1	6.79929	0.867648440449	1707	1714	-	ATTAGTTA
	MA1039.1	6.44284	0.859591426411	2157	2164	-	TTTTGGTG
	MA1039.1	6.27704	0.855843645899	860	867	+	ATTTGTTA
	MA1039.1	6.26484	0.855567936696	73	80	-	ATTTGGTC
	MA1039.1	6.17078	0.853441699227	1703	1710	-	GTTAGTTT
	MA1039.1	6.06621	0.851077983225	1826	1833	-	GTTAAGTG
	MA1039.1	5.82514	0.845628921723	1909	1916	+	AGTTGATG
	MA1039.1	5.82514	0.845628921723	1993	2000	-	AGTTGATG
	MA1039.1	5.79709	0.844994887561	854	861	-	ATTGGGTG
	MA1039.1	5.52714	0.838893059192	1024	1031	-	AGTGGTTG
	MA1039.1	5.17587	0.830953086836	2263	2270	-	GGTTTGTG
	MA1039.1	5.15255	0.830425995079	694	701	+	GGTTGGCG
	MA1039.1	4.86156	0.82384853061	2404	2411	+	AGCTGGTG
	MA1039.1	4.78474	0.822111939873	213	220	-	ATTAGTTT
	MA1039.1	4.68016	0.819748223871	940	947	-	ATTAAGTG
	MA1039.1	4.59955	0.817925988672	2460	2467	+	GTTTGGAG
	MA1039.1	4.52733	0.816293613426	489	496	-	GGTTAGTC
	MA1039.1	4.44629	0.814461817864	529	536	-	GTTGGTTA
	MA1039.1	4.44049	0.814330624259	603	610	+	TTTTGTTG

	MA1039.1	4.41022	0.813646395502	790	797	-	AGTAATTG
	MA1039.1	4.40121	0.81344273931	353	360	+	GATAGTTA
	MA1039.1	4.26249	0.810307134544	159	166	+	ATTTGTTT
	MA1039.1	4.26249	0.810307134544	1485	1492	+	ATTTGTTT
	MA1039.1	4.22398	0.809436775129	1458	1465	+	CGTGGGTA
	MA1039.1	4.21143	0.809153165424	185	192	+	AGTTGGAA
	MA1039.1	4.18351	0.808521879731	577	584	+	AGTCGTTG
	MA1039.1	4.13326	0.807386061287	1778	1785	+	ACTTGGTG
	MA1039.1	3.87896	0.80163794476	2294	2301	-	GATTGTTA
	MA1039.1	3.86676	0.801362235557	2267	2274	-	GATTGGTT
	MA1039.1	3.86507	0.801323950995	1422	1429	+	GTTTGGAA
P-*SmDXS3*	MA1039.1	12.6546	1.00000000264	2295	2302	-	GGTAGGTG
	MA1039.1	10.0118	0.940263411055	2453	2460	-	AGTTGGTA
	MA1039.1	8.91981	0.915580237479	84	91	-	GTTAGTTG
	MA1039.1	8.91981	0.915580237479	1034	1041	-	GTTAGTTG
	MA1039.1	8.91554	0.915483620697	8	15	+	GGTGGGTG
	MA1039.1	7.66308	0.887173405253	24	31	+	GTTTGTTA
	MA1039.1	7.44077	0.882148362517	2425	2432	+	CGTTGGTA
	MA1039.1	6.79502	0.867551823667	2472	2479	-	AGTGGGTA
	MA1039.1	6.41257	0.858907197653	489	496	-	AGTAAGTG
	MA1039.1	6.27704	0.855843645899	1189	1196	+	ATTTGTTA
	MA1039.1	6.06621	0.851077983225	2304	2311	-	GTTAAGTG
	MA1039.1	5.96066	0.848692354916	2447	2454	+	CGTAGTTA
	MA1039.1	5.70836	0.842989388734	1529	1536	+	TTTTGGTA
	MA1039.1	5.46816	0.837559997651	1667	1674	-	AGTAGGAG
	MA1039.1	5.46816	0.837559997651	1729	1736	-	AGTAGGAG
	MA1039.1	5.22975	0.832171014456	1991	1998	+	AATTGGTG
	MA1039.1	5.09066	0.829026894824	399	406	+	AGTTGATA
	MA1039.1	5.06178	0.828374127957	2429	2436	+	GGTAATTA
	MA1039.1	5.04958	0.828098418754	1533	1540	+	GGTAAGTT
	MA1039.1	4.78046	0.82201532309	2204	2211	-	AGTGGGTC
	MA1039.1	4.46214	0.814820142824	547	554	+	GGTTGATT
	MA1039.1	4.44049	0.814330624259	574	581	+	TTTTGTTG
	MA1039.1	4.44049	0.814330624259	1283	1290	-	CTTTGTTG
	MA1039.1	4.21143	0.809153165424	991	998	+	AGTTGGAA
P-*SmGGPPS1*	MA1039.1	7.99725	0.8947268997	1473	1480	+	AGTTGGTT
	MA1039.1	7.65089	0.88689769605	49	56	-	GTTTGGTT
	MA1039.1	7.38094	0.880795867681	1477	1484	+	GGTTGTTT
	MA1039.1	7.27637	0.878432151679	836	843	-	GGTTAGTG
	MA1039.1	6.96509	0.871396220961	2003	2010	+	TTTAGGTG
	MA1039.1	6.78709	0.867372731246	1454	1461	+	ATTAGGTT
	MA1039.1	6.44284	0.859591426411	202	209	+	TTTTGGTG

	MA1039.1	6.26484	0.855567936696	1552	1559	+	ATTTGGTT
	MA1039.1	5.70836	0.842989388734	601	608	-	TTTTGGTA
	MA1039.1	5.64853	0.841636893898	20	27	-	GTTTGTTC
	MA1039.1	5.64853	0.841636893898	2490	2497	-	GTTTGTTT
	MA1039.1	5.47877	0.837799718072	1274	1281	+	GTTTGATG
	MA1039.1	5.46816	0.837559997651	793	800	+	AGTAGGAG
	MA1039.1	5.33172	0.834475956326	1658	1665	+	GTTAAGTA
	MA1039.1	5.18077	0.831063855541	1474	1481	+	GTTGGTTG
	MA1039.1	5.15111	0.830393326017	60	67	-	GGATGGTG
	MA1039.1	4.96274	0.826135429588	2066	2073	+	TTTAGTTG
	MA1039.1	4.74429	0.821197680395	530	537	+	GTTTGATA
	MA1039.1	4.74429	0.821197680395	878	885	-	GTTTGATA
	MA1039.1	4.46214	0.814820142824	1340	1347	+	GGTTGATT
	MA1039.1	4.44049	0.814330624259	1221	1228	+	TTTTGTTG
	MA1039.1	4.44049	0.814330624259	1490	1497	+	TTTTGTTG
	MA1039.1	4.26249	0.810307134544	2155	2162	+	ATTTGTTT
	MA1039.1	4.10517	0.806751218752	860	867	-	GGTAGGAT
	MA1039.1	4.05957	0.805720575069	56	63	-	GGTGAGTG
	MA1039.1	3.94568	0.803146191583	545	545	-	ATTAAGTA
	MA1039.1	3.94568	0.803146191583	789	796	+	ATTAAGTA
	MA1039.1	3.8805	0.80167272637	594	601	-	ATTAGATA
P-*SmGGPPS3*	MA1039.1	11.2685	0.968670232503	1801	1808	+	AGTAGGTG
	MA1039.1	9.66544	0.932434196626	863	870	-	GTTTGGTA
	MA1039.1	7.99725	0.8947268997	900	907	+	AGTTGGTT
	MA1039.1	7.99725	0.8947268997	1960	1967	-	AGTTGGTT
	MA1039.1	7.38094	0.880795867681	904	911	+	GGTTGTTT
	MA1039.1	7.27637	0.878432151679	263	270	-	GGTTAGTG
	MA1039.1	6.96509	0.871396220961	1430	1437	+	TTTAGGTG
	MA1039.1	6.78709	0.867372731246	881	888	+	ATTAGGTT
	MA1039.1	6.26484	0.855567936696	979	986	+	ATTTGGTT
	MA1039.1	5.70836	0.842989388734	28	35	-	TTTTGGTA
	MA1039.1	5.64853	0.841636893898	1917	1924	-	GTTTGTTT
	MA1039.1	5.47877	0.837799718072	701	708	+	GTTTGATG
	MA1039.1	5.46816	0.837559997651	220	227	+	AGTAGGAG
	MA1039.1	5.33172	0.834475956326	1085	1092	+	GTTAAGTA
	MA1039.1	5.18077	0.831063855541	901	908	+	GTTGGTTG
	MA1039.1	4.96274	0.826135429588	1493	1500	+	TTTAGTTG
	MA1039.1	4.74429	0.821197680395	305	312	-	GTTTGATA
	MA1039.1	4.46214	0.814820142824	767	774	+	GGTTGATT
	MA1039.1	4.44049	0.814330624259	648	655	+	TTTTGTTG
	MA1039.1	4.44049	0.814330624259	917	924	+	TTTTGTTG
	MA1039.1	4.26249	0.810307134544	1582	1589	+	ATTTGTTT

	MA1039.1	4.10517	0.806751218752	287	294	-	GGTAGGAT
	MA1039.1	3.94568	0.803146191583	216	223	+	ATTAAGTA
	MA1039.1	3.8805	0.80167272637	21	28	-	ATTAGATA

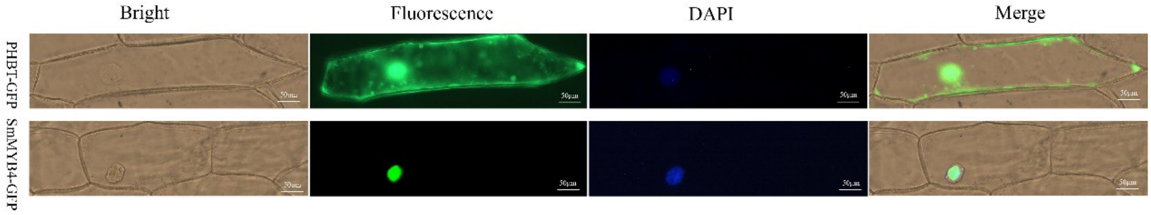

Figure S1. Subcellular localization of SmMYB4 in onion epidermal cells.

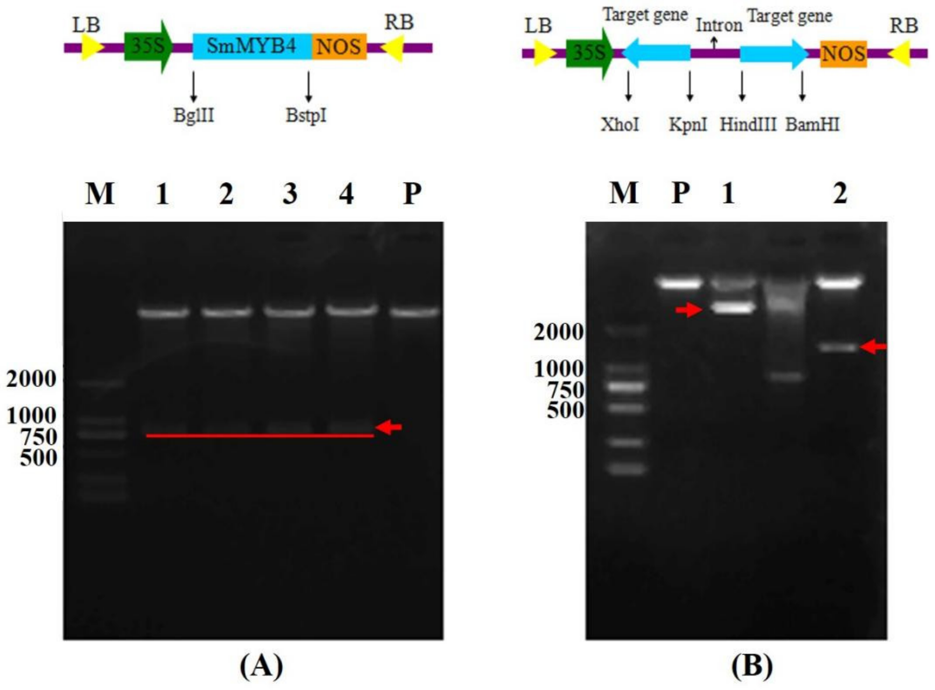

Figure S2. Restriction endonuclease digestion of the overexpression and RNAi expression vectors. A represents the Bgl II/BstP I digestion of the overexpression vectors, in which P represents plasmids, M refers to Marker DL2000, and 1-4 represents the digest results of Bgl II/BstP I. B represents the digestion of the RNAi expression vectors, in which P represents plasmids, M refers to Marker DL2000, 1 refers to the digestion of XhoI, and 2 refers to the digestion of Not I. The target fragment is marked with red line or arrows.

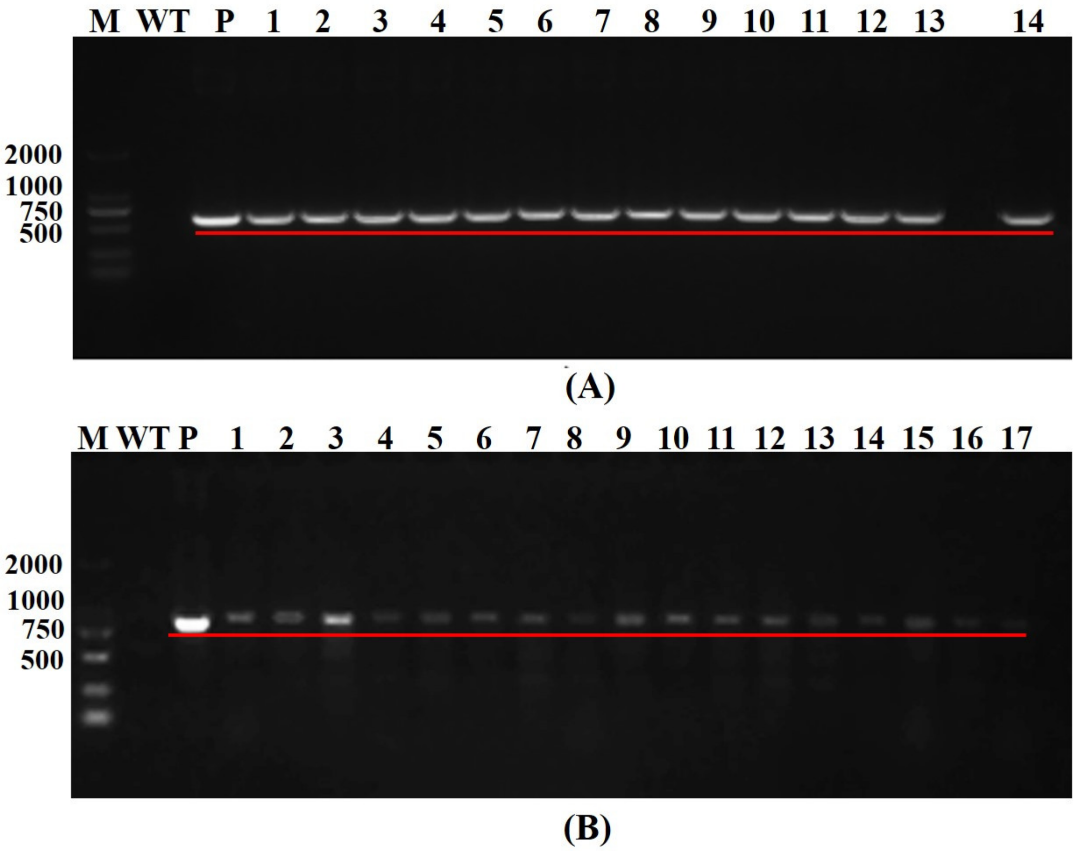

Figure S3. Detection of the transgenic lines at the DNA level Detection. Results of the overexpression and interference transgenic lines were individually showed in Figure S3A,B. P indicates positive control (Plasmids), WT indicates negative control (untransformed plants), and M indicates Marker DL2000.

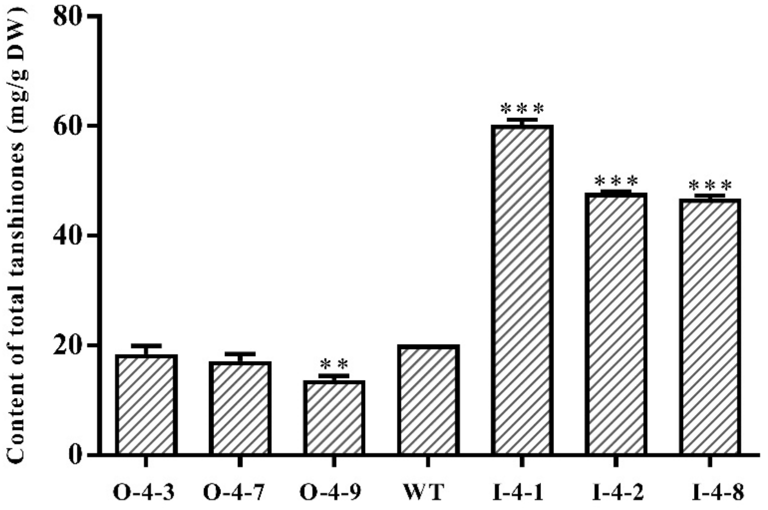

Figure S4. Total tanshinone contents in the roots of transgenic plants (**p<0.01, ***p<0.001).
